# Light Sheet Illumination for 3D Single-Molecule Super-Resolution Imaging of Neuronal Synapses

**DOI:** 10.3389/fnsyn.2021.761530

**Published:** 2021-11-24

**Authors:** Gabriella Gagliano, Tyler Nelson, Nahima Saliba, Sofía Vargas-Hernández, Anna-Karin Gustavsson

**Affiliations:** ^1^Department of Chemistry, Rice University, Houston, TX, United States; ^2^Applied Physics Program, Rice University, Houston, TX, United States; ^3^Smalley-Curl Institute, Rice University, Houston, TX, United States; ^4^Systems, Synthetic, and Physical Biology Program, Rice University, Houston, TX, United States; ^5^Institute of Biosciences & Bioengineering, Rice University, Houston, TX, United States; ^6^Department of Biosciences, Rice University, Houston, TX, United States; ^7^Laboratory for Nanophotonics, Rice University, Houston, TX, United States

**Keywords:** 3D single-molecule imaging, super-resolution microscopy, light sheet illumination, point spread function engineering, neuronal synapses

## Abstract

The function of the neuronal synapse depends on the dynamics and interactions of individual molecules at the nanoscale. With the development of single-molecule super-resolution microscopy over the last decades, researchers now have a powerful and versatile imaging tool for mapping the molecular mechanisms behind the biological function. However, imaging of thicker samples, such as mammalian cells and tissue, in all three dimensions is still challenging due to increased fluorescence background and imaging volumes. The combination of single-molecule imaging with light sheet illumination is an emerging approach that allows for imaging of biological samples with reduced fluorescence background, photobleaching, and photodamage. In this review, we first present a brief overview of light sheet illumination and previous super-resolution techniques used for imaging of neurons and synapses. We then provide an in-depth technical review of the fundamental concepts and the current state of the art in the fields of three-dimensional single-molecule tracking and super-resolution imaging with light sheet illumination. We review how light sheet illumination can improve single-molecule tracking and super-resolution imaging in individual neurons and synapses, and we discuss emerging perspectives and new innovations that have the potential to enable and improve single-molecule imaging in brain tissue.

## Introduction

**Figure 1:**
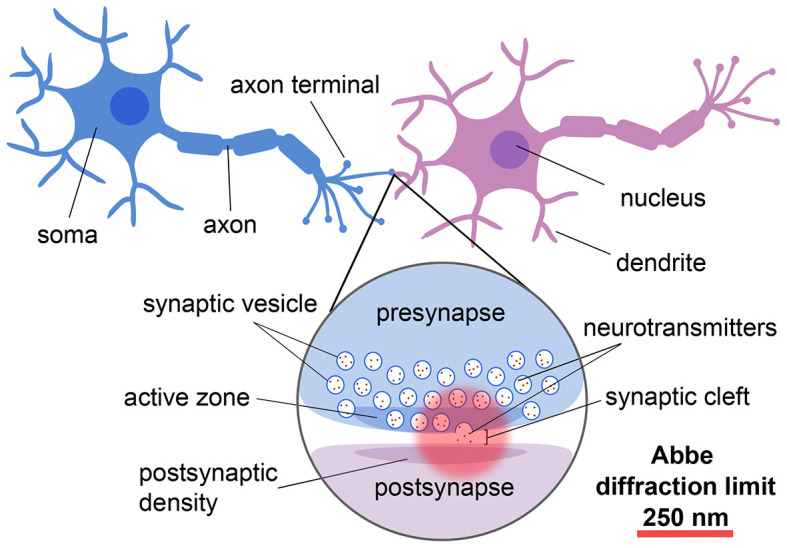
Schematic showing the different components and length scales involved in neuronal and synaptic imaging demonstrating the importance of super-resolution techniques for resolving these structures.

Neurons are the specialized units of the nervous system that communicate *via* the release of chemical neurotransmitters at the junctions, or synapses, between them (for reviews, see e.g., Guillery, 2005; Yuste, 2015; Figure 1). Given that neurons and synapses are intricate and that the width of the synaptic cleft is on the order of tens of nanometers, advanced techniques are needed to image and understand their architecture and molecular dynamics at the nanoscale.

Early methods of studying neuronal tissues include electron microscopy (EM), which was used to directly visualize the synapse. EM studies allowed measurements of the synaptic cleft (De Robertis and Bennett, 1955; Palay and Palade, 1955), and discoveries of other distinct features of neuronal communication, such as the accumulation of synaptic vesicles (SVs) at presynaptic terminals (De Robertis and Bennett, 1954; Palade, 1954). This discovery in combination with the hypothesis of neurotransmitter release (Del Castillo and Katz, 1954) eventually led to the conclusion that synaptic vesicles were the mechanism by which neurotransmitters were stored and released across the synapse (De Robertis et al., 1963). Hence, with the dawn of EM came the ability to investigate the sub-cellular organization of synapses at exquisite spatial resolution (for reviews, see e.g., Siksou et al., 2009; Harris and Weinberg, 2012). However, EM is limited in its ability to study molecular assembly and mechanisms because of restricted labeling specificity, poor temporal resolution, and the necessity to work with dead samples.

Fluorescence microscopy, on the other hand, offers the benefits of very specific labeling, excellent temporal resolution, and the ability to study live samples. However, due to the diffraction limit imposed by the finite wavelength of light, conventional optical microscopy has been inherently limited in its ability to resolve cellular nanoscale structures (Abbe, 1873). This issue was overcome by the invention of super-resolution (SR) fluorescence microscopy over a decade ago and marked an important milestone in imaging technology, which was recognized with the Nobel Prize in Chemistry 2014 to W. E. Moerner (Moerner, 2015), Eric Betzig (Betzig, 2015), and Stefan Hell (Hell, 2015). Since its invention, SR microscopy has paved the way for detailed studies of synaptic architecture and its molecular mechanisms and dynamics. An emerging approach that shows great promise for imaging in biological samples and has recently been implemented for single-molecule tracking and SR imaging is light sheet illumination (for reviews, see e.g., Power and Huisken, 2017; Gustavsson et al., 2018b). After introducing light sheet illumination and a comparison with other conventional illumination strategies, we will briefly review the history and application of deterministic optical SR techniques for neuronal imaging, as well as the fundamentals and applications of single-molecule tracking and SR imaging in 2D and 3D. We will then discuss light sheet illumination’s impact on the improvement of these techniques and its relevance to studies of neurons and synapses. We will conclude with some emerging perspectives that have the possibility to further improve these methods and lead to new discoveries about the function of synapses at the molecular level.

## Light Sheet Illumination for Optical Sectioning in Thick Samples

Light sheet fluorescence microscopy (LSFM), also known as selective plane illumination microscopy (SPIM), is a wide-field method where the sample is illuminated with a thin sheet of light introduced perpendicular to the detection axis (Huisken et al., 2004). This method optically sections the sample and excites fluorophores only in a thin slice around the image plane. This results in reduced fluorescence background, photobleaching, and photodamage, which makes LSFM a great option for imaging of thick and sensitive samples.

The idea of implementing a sheet of light as an illumination mechanism was initially presented in 1902 (Siedentopf and Zsigmondy, 1902), where sunlight projected through a slit aperture was utilized to observe gold nanoparticles. Light sheet illumination then became a powerful contribution to the scientific community in the 1990s when it was combined with fluorescence microscopy, as it allowed researchers to image biological processes in 3D. Specifically, a form of LSFM called orthogonal-plane fluorescence optical sectioning (OPFOS; Voie et al., 1993) was the first to use a cylindrical lens to create a light sheet and was developed to image the internal 3D architecture of the cochlea. Another form of LSFM, a thin light-sheet microscope (TLSM), was developed to aid oceanographers in observing aquatic microbes (Fuchs et al., 2002) before the subsequent development of the updated design SPIM (Huisken et al., 2004). SPIM was originally developed to allow for non-invasive imaging of live embryos where the sample could be rotated for the sequential acquisition of multiple views (Huisken et al., 2004). Since then, light sheet technologies have advanced rapidly to achieve enhanced image quality, axial resolution, field-of-view (FOV) size, and acquisition rates (for a review, see e.g., Gustavsson et al., 2018b), and LSFM has become the gold standard for 3D and 4D imaging of developmental processes and live species behavior (for reviews, see e.g., Huisken and Stainier, 2009; Santi, 2011; Power and Huisken, 2017).

Light sheet illumination has several benefits over more conventional illumination strategies. One of the most commonly used illumination strategies for fluorescence imaging is wide-field epi-illumination, where the entire sample is illuminated at once and the fluorescence light is detected through the same objective as is used for illuminating the sample. Although straightforward to implement, this approach results in increased fluorescence background due to excitation of fluorophores away from the image plane, greater risk of premature photobleaching of fluorophores, and increased risk of photodamaging sensitive live samples. These factors are also critical when it comes to single-molecule imaging: the increased background leads to reduced precision in localizing single molecules, and the premature photobleaching of fluorophores outside of the current detection volume reduces the density of localizations, which effectively reduces the achievable resolution in the reconstruction. We will discuss these considerations in more detail in later sections.

Confocal microscopy is another commonly used approach for fluorescence imaging that provides background reduction through the use of a pinhole that blocks light originating from planes away from the image plane. However, its point-scanning nature makes it a much slower approach than wide-field alternatives. The speed of the acquisition can be improved using spinning disk confocal imaging, where the confocal concept is parallelized using an array of pinholes on a rotating disk. This approach has been used together with single-molecule imaging (Hosny et al., 2013; Chen X. et al., 2015). Even though the light is only detected near the image plane, the excitation light still passes through the entire sample both in conventional confocal and spinning disk confocal microscopy, which increases the risk of photobleaching and photodamage. The issue with premature photobleaching of fluorophores outside of the current detection volume has been mitigated by pairing spinning disk confocal imaging with DNA-PAINT (Schueder et al., 2017), where fluorophores are continuously replenished from a large, diffusing pool. However, both the excitation intensity and the detection efficiency can be limited by the disk, which reduces the localization precision for single-molecule imaging.

Total internal reflection fluorescence (TIRF) microscopy, also known as evanescent wave microscopy, is a wide-field approach that exploits the evanescent wave resulting from a laser beam that is totally internally reflected at the interface between the coverslip and the sample (Axelrod, 2001). The evanescent wave reaches a few hundred nanometers into the sample and thus TIRF provides exquisite optical sectioning with very low fluorescence background, photobleaching, and photodamage. TIRF has therefore been used extensively for single-molecule imaging (Bates et al., 2007; Shroff et al., 2007). However, it is limited to imaging very close to the coverslip and cannot be used for imaging deeper into the sample. In comparison, the optical sectioning capability of LSFM efficiently reduces the issues of high background fluorescence, photobleaching, and photodamage that are problematic in the other illumination strategies. LSFM is also a wide-field technique that is compatible with volumetric imaging of thick samples away from the coverslip.

For these reasons, LSFM has successfully been used for numerous applications in large-scale imaging, including imaging of the brain (for a review, see e.g., Corsetti et al., 2019). Specifically, LSFM has been applied to large neuronal populations such as those of the vomeronasal organ of the mouse (Holekamp et al., 2008) and to whole-brain imaging in mice (Dodt et al., 2007; Mertz and Kim, 2010), rats (Stefaniuk et al., 2016), songbirds (Rocha et al., 2019), and in zebrafish larvae to detect rapid changes in neural activity (Ahrens et al., 2013; Panier et al., 2013; Vladimirov et al., 2014; Park et al., 2015; Quirin et al., 2016; Greer and Holy, 2019). LSFM has also been improved to enable fast imaging of transient events in rat dendritic tissue (Haslehurst et al., 2018), to investigate the arrangement of human neural aggregates and their Ca^2+^ oscillations (Gualda et al., 2014), to study the interaction between sensory neurons and Schwann cells during neurotrauma (Xiao et al., 2015), and for functional volumetric imaging of hippocampal neurons in a 3D culture system (Chen et al., 2019). LSFM has also been paired with deep neural networks for imaging neurons in transgenic mouse brains (Zhao et al., 2020).

Overall, LSFM serves a very important role in imaging the brain as our understanding of the interactions among large neural networks depends upon the communication of multiple neurons across vast areas. Furthermore, LSFM provides the gentle illumination required for live-cell imaging and therefore, when combined with single-particle tracking (SPT) and single-molecule SR methods, offers great potential to study structures and interactions in synapses at the nanoscale. In the following sections, different SPT and SR methods will be described, in addition to the ways in which they can be improved by pairing with LSFM.

## Deterministic Super-Resolution Techniques and Their Applications in Neuronal and Synaptic Imaging

There are two major groups of optical SR methods: stochastic and deterministic. We will discuss stochastic methods relying on single-molecule localization in some detail in the later sections. In deterministic SR imaging, knowledge of the spatial distribution of the excitation light is utilized in combination with the non-linear response of fluorophores to excitation as a means to circumvent the diffraction limit.

Stimulated emission depletion (STED) microscopy is an SR technique that utilizes a doughnut-shaped depletion laser overlaid atop a confocal excitation spot to deplete fluorophores in the periphery of the target region, effectively reducing the extent of the confocal spot by stimulating transitions of fluorophores from an excited singlet to the ground singlet state (Hell and Wichmann, 1994; Sahl et al., 2017). For more detailed information on STED microscopy, see e.g., Eggeling et al. (2013), Hell (2015), and Blom and Widengren (2017). STED has been used successfully for live-cell imaging, as demonstrated in *S. cerevisiae* and *E. coli* over two decades ago (Klar et al., 2000). STED has also been applied extensively to study neurons and synapses. In one example, STED was used to resolve individual vesicles in the synapse and confirm that the vesicle membrane protein synaptotagmin I clusters in patches on the presynaptic membrane independently of nerve terminal stimulation (Willig et al., 2006). Furthermore, two-color STED live-cell imaging was used to investigate the ultrastructure of endogenous F-actin in hippocampal neurons and revealed a subcortical periodic actin lattice in both axons and dendrites (D’Este et al., 2015). Live-cell STED imaging was also used to image the structure and morphological plasticity of dendritic spines in hippocampal samples and brain slices (Nägerl et al., 2008; Tønnesen et al., 2011; Urban et al., 2011). More recently, STED microscopy has been utilized to reveal activity-dependent enlargement in presynaptic boutons and axon shafts (Chéreau et al., 2017). However, despite its contributions to the field, STED is limited by its point-scanning nature, which makes it non-ideal for imaging over larger fields of view or for tracking of single-molecule dynamics. Given the high-power densities needed, there is also an increased risk for premature photobleaching and phototoxicity, which potentially limits its use for live-cell imaging of sensitive samples. Because absorption of biological material is minimal in the far-red region, far-red emitting fluorescent proteins have been used to mitigate the risk of photodamaging samples during STED imaging of dendritic spines (Wegner et al., 2017). However, such fluorescent proteins typically display lower photostability and quantum yield relative to shorter wavelength fluorescent proteins. The use of novel far-red synthetic dyes, such as silicon rhodamines (SiR; Lukinavičius et al., 2013, 2014; D’Este et al., 2015), in combination with Halo- or SNAP-tag labeling is another approach that holds great promise to further improve imaging in live cells in future STED studies. The issue of photobleaching was mitigated with the development of a technique called super-resolution shadow imaging (SUSHI; Tønnesen et al., 2018). In this method, extracellular fluid in the brain is fluorescently labeled and imaged using 3D-STED, creating a negative image of the structures to be studied and thus reducing the impact of photobleaching. SUSHI is well-suited for visualizing the structure of synapses since the synaptic clefts are full of fluorescently labeled extracellular fluid (Hrabetova et al., 2018). Even though SUSHI is limited in that it cannot resolve single-molecule mechanisms or be used to study specific structures, it can be combined with single-molecule localization approaches to correlate structural context with molecular specificity (Inavalli et al., 2019).

Reversible saturable optical fluorescence transitions (RESOLFT) microscopy is a more general term for techniques which make use of photoswitchable molecules and various types of on and off states together with inhomogeneous illumination (Schwentker et al., 2007). STED is one example within the RESOLFT concept, but other RESOLFT methods utilize switching between other types of states, such as an excited singlet and dark triplet states, where lower laser intensities are sufficient for switching. These other methods within the RESOLFT concept can thus be gentler for live-cell imaging. For example, RESOLFT has been used to image dendritic spines with low light intensities in 2D in living brain slices (Testa et al., 2012) and in 3D together with the imaging of the postsynaptic protein Homer1 in cultured neurons (Bodén et al., 2021). A variation of RESOLFT called molecular nanoscale live imaging with sectioning ability (MoNaLISA) allows intrinsic optical sectioning of large samples and has been demonstrated for imaging in living neurons and brain tissue (Masullo et al., 2018).

Another method that utilizes knowledge of the spatial distribution of the excitation illumination to circumvent the diffraction limit is structured illumination microscopy (SIM), where the effective lateral resolution is improved over the classical diffraction limit by a factor of two (Gustafsson, 2000). Because SIM requires relatively low excitation intensities, it is gentle and thus favorable for live-cell imaging. It is also compatible with multi-color imaging. Since it is a wide-field technique, in contrast to STED, SIM allows for imaging of large fields of view with good temporal resolution. For more detailed reviews, see e.g., Hirano et al. (2015), Heintzmann and Huser (2017), and Zheng et al. (2021). SIM has been used to image many different challenging cell types, including neurons. For example, it was used to study the plasticity of dendritic spines in mice hippocampal neurons (Guo et al., 2018). SIM was also used to reveal that PDZD8, an ER protein, was localized at ER-mitochondria contact sites in mammalian neurons and regulated synaptically-evoked Ca^2+^ dynamics (Hirabayashi et al., 2017). SIM studies in neuronal growth cones also aided in the discovery of a distinct form of endocytosis at the leading edge responsible for coordinated vesicle and actin-bundling generation during axon growth (Nozumi et al., 2017). Additionally, SIM was used to determine the spatial distributions of the presynaptic protein synapsin and the postsynaptic proteins Homer1 and PSD-95 through imaging of thousands of synapses (Lagache et al., 2018). SIM can be generalized to 3D by generating a pattern along both the lateral and axial directions (Gustafsson et al., 2008). 3D SIM revealed complex actin structures in the neuron growth cone and was used to observe the dynamics of cortical actin in hippocampal neurons and glia (Fiolka et al., 2012). While classical SIM most definitely continues to prove itself useful for neuronal imaging, it presents limitations in that the technique only offers a two-fold improvement in resolution. Non-linear SIM (NL-SIM), or saturated SIM (SSIM), however, exceeds this limit by making use of saturating excitation intensities (Gustafsson, 2005). Theoretically, this non-linear method can achieve infinite resolution and, like classical SIM, it can be expanded to 3D, but in practice, the resolution achieved is typically limited to around 50 nm (Gustafsson, 2005). While such a technique is advantageous in that it allows for improved resolution in 3D, it requires high laser intensities to nearly reach saturation conditions, which increases the rate of photobleaching. Hence, SSIM requires samples to be labeled with bright and photostable fluorophores (Gustafsson, 2005). SSIM also requires acquisition with multiple patterns, which limits the temporal resolution.

Deterministic SR techniques each offer their own unique advantages and will continue to provide important contributions to our understanding of neuronal and synaptic function. However, they are not single-molecule techniques, and they are limited to studies of molecular mechanisms and interactions. In the rest of this review, we will focus on techniques that allow for the fundamental goal of detecting single molecules in 2D and 3D, and how these techniques further benefit from the combination with light sheet illumination for imaging of neurons and synapses.

## Single-Molecule Localization Microscopy

**Figure 2:**
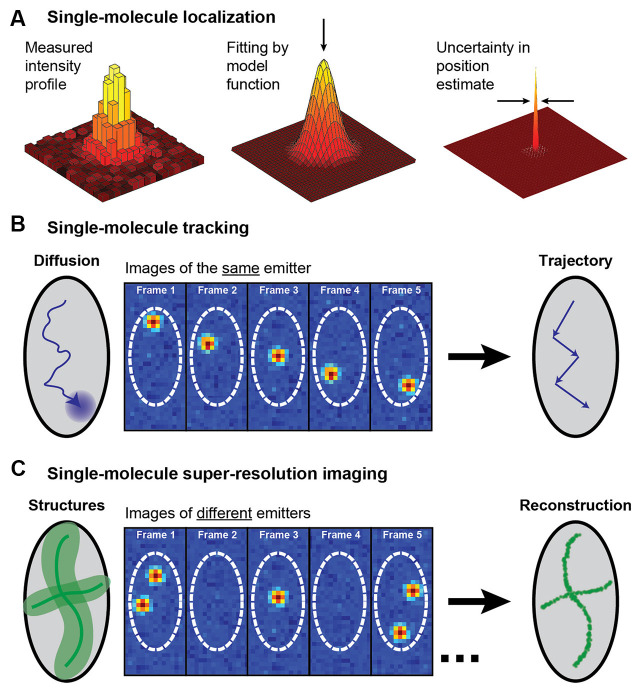
Schematic illustrating single-molecule super-localization, tracking, and super-resolution imaging. **(A)** The noisy diffraction-limited pattern of an isolated fluorophore’s intensity profile on the camera can be fit with a model function, such as a 2D Gaussian, to estimate the position of the fluorophore with a precision that is better than the diffraction limit. **(B)** In single-molecule tracking, the same single fluorophore is super-localized over multiple frames to create a trajectory that yields information about the fluorophore dynamics. **(C)** A diffraction-limited image with all molecules fluorescing simultaneously can be super-resolved by sequentially super-localizing many different spatially isolated molecules. This approach requires some active control to allow just a small fraction of the fluorophores to emit in each camera frame. Figure reprinted with permission from von Diezmann et al. (2017). Copyright (2017) American Chemical Society.

In this review we will consider two applications of single-molecule localization microscopy: SPT of the motion of individual molecules to acquire information about their dynamics and interactions, and single-molecule SR microscopy for resolving static extended structures (for reviews, see e.g., von Diezmann et al., 2017; Möckl and Moerner, 2020; Figure 2). Both of these applications rely on the detection of the position of single molecules and can be coupled with light sheet illumination to better understand structures at the nanoscale. In localization microscopy, a single fluorophore is localized by detecting a sufficient number of photons from the fluorophore on a camera and then analyzing the diffraction-limited spatial distribution of photons, known as the point spread function (PSF). Each photon in the measured PSF can be treated as a sample of the probability distribution centered on the true position of the fluorophore, and an estimator of the center of the PSF can then be used to localize the fluorophore position in 2D with a precision that is much finer than the width of the intensity distribution of the PSF. In the following sections, we will describe methods for SPT and single-molecule SR imaging and showcase some examples of their applications for imaging of neurons and synapses. For extensive reviews of applications of these techniques in neuroscience, see e.g., Kim et al. (2010), Maglione and Sigrist (2013), Tønnesen and Nägerl (2013), Bannai (2018), Nosov et al. (2020), Carvalhais et al. (2021), Werner et al. (2021), and Zieger and Choquet (2021).

## Single-Particle Tracking in 2D Reveals Information About Molecular Dynamics and Interactions

SPT is a method used to observe the dynamics and intermolecular interactions of individual particles and molecules at high spatial and temporal resolution (for a review, see e.g., Shen et al., 2017). In SPT, the same particle is localized and tracked over time, providing information on nanoscale dynamics and interactions beyond the optical diffraction limit. With the ability to directly monitor individual particles, one can obtain information about heterogeneous systems and unique events that would have otherwise been lost in averaged measurements. SPT can therefore provide a more complete understanding of the behavior of individual molecules in complex systems and of the mechanisms behind various biological processes.

For this reason, SPT has been used extensively to study nanoscale dynamics in neurons and synapses. Quantum dot (QD)-SPT is a commonly used technique for observing the molecular membrane dynamics of neurons, and has led to insights into the functions of neurotransmitter transporters (Thal et al., 2019) and receptors (Ehrensperger et al., 2007; Bürli et al., 2010; Arizono et al., 2012). Despite the discoveries QD-SPT has contributed to neuroscience, there are drawbacks associated with using QDs. The large size of QDs compared to organic dyes or fluorescent proteins limits the mobility of the molecule in narrow areas such as the synaptic cleft (Groc et al., 2007; Alcor et al., 2009). Additionally, QDs have to be tagged to the proteins of interest and blink erratically between on and off states which complicates their use in SPT (Groc et al., 2007; Alcor et al., 2009).

**Figure 3:**
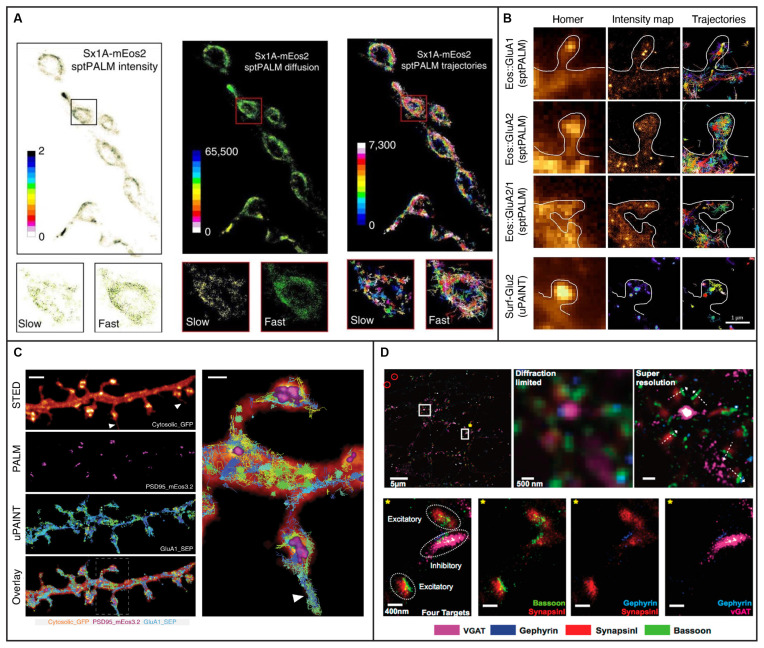
Examples of 2D single-molecule tracking and 2D super-resolution imaging in neurons and synapses. **(A)** sptPALM was used to image the distribution and mobility of attachment receptor protein syntaxin1A fused with photoconvertible fluorescent protein mEos2 in the motor nerve terminal of *Drosophila* larvae. Insets demonstrate average intensity, diffusion coefficient, and trajectory map showing slow and fast populations of syntaxin1A on a synaptic bouton. Figure reprinted from Bademosi et al. (2016). Reprinted with permission from Springer, under Creative Commons Attribution 4.0 International License. **(B)** The organization of AMPA receptors into nanodomains inside the spines of live hippocampal neurons demonstrated with single-molecule tracking techniques sptPALM and uPAINT. The first column shows diffraction-limited images of Homer1c and the following two columns show intensity maps and trajectories from measurements using sptPALM (top three rows) or uPAINT (bottom row). The scale bar is 1 μm. Adapted from Nair et al. (2013) (https://www.jneurosci.org/content/33/32/13204). **(C)** Combining deterministic approaches STED and SUSHI with stochastic techniques PALM and uPAINT enabled correlative super-resolution imaging of neuron morphology and analysis of the distribution and dynamics of synaptic proteins in live hippocampal neurons. The right panel is a close up of the area marked with a rectangle in the overlay. The scale bar is 2 μm in the left panel and 500 nm in the close up. Reprinted by permission from Springer Nature: Springer Nature, Nature Methods, (Inavalli et al., 2019), copyright 2019. **(D)** The four synaptic proteins vGAT, Gephyrin, SynapsinI, and Bassoon imaged sequentially with Exchange-PAINT. The top left panel shows a merged image of the synaptic proteins, where gold nanoparticles were used as fiducials for registration (circled in red). The scale bar is 5 μm. The top middle and top left panels show diffraction-limited and super-resolved images, respectively, of the region in the top left panel marked with a white square without a star. The super-resolved image allows distinction of the orientation of individual synapses as shown with white arrows. The scale bar is 500 nm. The bottom panels show the region marked with a white square and yellow star in the top left panel. The four targeted synaptic proteins are first shown together and then pair-wise. The scale bars are 400 nm. Reprinted with permission from Wang et al. (2017). Copyright (2017) American Chemical Society.

SptPALM is a method that overcomes some of the challenges presented by QD-SPT by combining SPT with (fluorescence) PhotoActivation Localization Microscopy [(f)PALM], where photoactivatable fluorophores are activated and then tracked until they photobleach (Manley et al., 2008). SptPALM utilizes smaller labels that also typically lessen the issues with blinking and are well suited for live-cell imaging. Thousands of these photoactivated fluorophores, most commonly fluorescent proteins, can be tracked simultaneously in live cells by single-molecule localization followed by trajectory reconstruction, allowing for studies of high-density dynamics of single molecules (Manley et al., 2008, 2010). SptPALM in neurons has provided insight into the organization and dynamics of individual actin molecules within dendritic spines (Tatavarty et al., 2009; Frost et al., 2010), revealing their heterogenous distribution and role in supporting diverse processes in the synapse. SptPALM has also revealed a heterogeneous distribution of CaMKII in dendritic spines in non-stimulated and stimulated rat hippocampal neurons, suggesting that CaMKII fulfills multiple functions both inside and outside of the postsynaptic density (Lu et al., 2014). Another study has also revealed that CaMKII has both a kinase- and structure-dependent role for actin remodeling in the spine (Kim et al., 2015). In addition, sptPALM has been used to track the dynamics of the transcription factor NF-κB p65 (Widera et al., 2016), which is transported from the synapse to the nucleus upon glutamate activation. Another study using sptPALM investigated the mobility of syntaxin1A, a protein involved in synaptic vesicle docking, and it was found that the mobility increased in response to opto- and thermo-genetic neuronal stimulation and that diffusion and trapping of syntaxin1A in nanoclusters regulated neurotransmitter release (Bademosi et al., 2016; Figure 3A). Additionally, sptPALM was used to investigate the effect of Shank knockdown on the mobility of cortactin and revealed that Shank proteins are key intermediates between the synapse and the spine actin cytoskeleton *via* cortactin (MacGillavry et al., 2016). Furthermore, sptPALM has been used to explore the mechanisms connecting voltage-gated calcium channels with short-term plasticity (Heck et al., 2019), as well as the spatiotemporal distribution of postsynaptic AMPA receptors (Hoze et al., 2012; Nair et al., 2013). SptPALM has also been used to map how the membrane dynamics of GABAA receptors are altered with mutations associated with epilepsy (Bouthour et al., 2012). These examples of SPT in neurons and synapses highlight the versatility and strength of SPT for improving our understanding of the function of synapses at the molecular level. However, as we will discuss further in later sections, such methods for neuronal imaging can be further improved upon with the incorporation of LSFM.

## Single-Molecule Super-Resolution Microscopy in 2D Unveils Structures at The Nanoscale

The second application of single-molecule localization microscopy, single-molecule SR imaging, strives to map nanoscale extended structures that are densely labeled with fluorophores. In addition to being able to localize the positions of the single molecules, localization-based SR imaging also requires some form of control of the density of the fluorophores that emit in each camera frame. Various methods have been developed using some photophysical, photochemical, or binding and unbinding mechanism to keep most of the fluorophores in an off state to ensure that just a small, non-overlapping subset of the fluorophores fluoresce simultaneously. By localizing different fluorophores in many subsequent frames, a point-by-point reconstruction of the underlying structure can be created. Methods that utilize single-molecule localization microscopy to achieve SR include (f)PALM (Betzig et al., 2006; Hess et al., 2006), (direct) Stochastic Optical Reconstruction Microscopy (d STORM; Rust et al., 2006; Heilemann et al., 2008), and various methods based on Point Accumulation for Imaging in Nanoscale Topography (PAINT; Sharonov and Hochstrasser, 2006). For more detailed reviews about these stochastic SR methods, please see e.g., von Diezmann et al. (2017), Möckl and Moerner (2020), and Lelek et al. (2021).

These stochastic SR methods have been used for numerous 2D studies of the neuronal synapse. For example, PALM has been used to study the spatial distribution of perisynaptic actin and its correlation with the postsynaptic density proteins GKAP and PSD-95 (Frost et al., 2010), to quantify the morphology of dendritic spines (Izeddin et al., 2011), and to reveal that nanoscale scaffolding domains within the postsynaptic density concentrate synaptic AMPA receptors (MacGillavry et al., 2013).

Two- or three-color STORM has been applied in conjunction with large-volume, automated, ultrathin sectioning to image ganglion cells (Sigal et al., 2015), to image parvalbumin-positive interneurons and their associated extracellular matrix, called perineural nets, in mouse primary visual cortices (Sigal et al., 2019), to discover a spatial correlation between AMPA receptor nanodomains and the post-synaptic adhesion protein neuroligin-1 (Haas et al., 2018), and to determine the nanoscale co-organization of AMPA receptors, NMDA receptors, and mGluR at excitatory synapses (Goncalves et al., 2020). DSTORM has also been combined with PALM to enable two-color imaging for studies of the spatial relation between actin in dendritic spines and the postsynaptic density protein Shank2 (Izeddin et al., 2011), with sptPALM to map the plasma membrane in primary hippocampal rat neurons (Ries et al., 2012), and with confocal approaches to investigate NMDA-receptor activation at single synapses (Metzbower et al., 2019).

DNA-PAINT (Jungmann et al., 2010) is a powerful implementation of PAINT where short single-stranded DNA oligonucleotides tagged with a fluorophore transiently bind to complementary oligonucleotides which are bound to a target molecule, such as an antibody to a protein of interest. DNA-PAINT overcomes PAINT’s limitations of target selectivity and specificity and, like PAINT, is not limited by photobleaching since the binding is reversible and bleached fluorophores can be replaced by an excess of unbleached ones. Since the on/off switching is controlled by the choice of oligonucleotide sequences rather than by the photophysical or photochemical properties of the fluorophore, DNA-PAINT also allows for a wider selection of fluorophores than (f)PALM and (d)STORM. An additional benefit is that multiplexing can be done through the use of multiple different oligonucleotide pairs imaged sequentially using the same fluorophore—a method called Exchange-PAINT (Jungmann et al., 2014). Exchange-PAINT mitigates the issue of chromatic-aberration induced offsets between different color channels that arise when using other techniques where different fluorophores are used. A tradeoff is that DNA- and Exchange-PAINT imaging are typically much slower than the other single-molecule SR methods. The recently developed Peptide-PAINT (Eklund et al., 2020) makes use of small, programmable peptide pairs instead of the single-stranded DNA oligonucleotides used in DNA-PAINT. In addition to their smaller size, these peptides can produce more favorable kinetics than their oligonucleotide counterparts and can, in that way, improve the imaging speed. DNA-PAINT conventionally requires the target cell to be fixed and permeabilized, meaning this method is typically not compatible with live-cell imaging. LIVE-PAINT (Oi et al., 2020) addresses this issue by combining peptides for labeling with fluorescent proteins which are coded for and expressed within the target cell. Quantitative DNA-PAINT (qPAINT) is a PAINT approach that allows for counting of the number of targets (Jungmann et al., 2016), and it has been utilized e.g., to image and estimate copy numbers of surface AMPA-type receptors at synapses of rat hippocampal neurons (Böger et al., 2019). In another study, Universal (u)PAINT (Giannone et al., 2010), which utilizes the PAINT concept for tracking of dynamics in membranes, was used together with sptPALM, dSTORM, STED, and EM to investigate the correlation between the dynamics and distribution of AMPA receptors with the position of clusters of the postsynaptic density protein PSD-95 (Nair et al., 2013; Figure 3B). Another recent study combined uPAINT with STED and PALM, and SUSHI with sptPALM and PALM, to study the position and movements of synaptic proteins within the morphological context of growth cones and dendritic spines (Inavalli et al., 2019; Figure 3C). UPAINT has also been combined with PALM for tracking of transmembrane proteins over postsynaptic densities whose internal structures were simultaneously super-resolved. The results provided important experimental confirmation that the density of scaffold proteins in the postsynaptic density strongly influences the mobility of transmembrane proteins (Li and Blanpied, 2016). Furthermore, uPAINT was combined with sptPALM to investigate the degree to which the mobility of AMPA receptors depends on protein crowding in the synapse (Li et al., 2016). Exchange-PAINT has also been demonstrated for up to eight-target imaging in primary neurons and included the co-localization of the four synaptic proteins Bassoon, Synapsin1, Gephyrin, and vGAT (Wang et al., 2017; Figure 3D).

All these stochastic SR techniques can be improved upon further by the combination with LSFM for optical sectioning. Some important technical considerations on improving both SPT and single-molecule SR imaging will be described in the next section.

## Considerations for Improving Single-Molecule Tracking and Super-Resolution Imaging

In this section, we will provide some technical details on how to improve the achievable localization precision which may prove helpful for the user. These considerations will also highlight the benefits of using LSFM for SPT and stochastic SR imaging.

The precision that can be achieved from single-molecule localization microscopy depends on multiple parameters, such as the number of the detected signal and background photons, the effective pixel size of the images, and the choice of position estimator. The simplest position estimator is the centroid or average photon position, but superior estimators are preferable. A simple 2D function such as a Gaussian or Airy function may be used as a model of the PSF, in combination with a fitting criterion such as a least squares or maximum likelihood estimator (MLE). MLE can provide improved localization precision compared to least-squares Gaussian fitting, especially for low background levels, but at the cost of computational complexity (Rieger and Stallinga, 2014). A common choice that balances analysis speed and acceptable precision is to fit using a 2D Gaussian with a constant background offset and an unweighted least squares estimator (Mortensen et al., 2010). For extensive reviews on the merits of different analysis approaches, see e.g., Abraham et al. (2009), Deschout et al. (2014), and Small and Stahlheber (2014).

Two other ways to improve the localization precision are to increase the signal photons from the target fluorophores and to reduce the background fluorescence coming from the rest of the sample. The first steps to achieve a reduction of background from the rest of the sample are to ensure proper filtering of Rayleigh and Raman scattered light, shield from light from other sources in the room, use specific labels, and work with far-red fluorophores where the autofluorescence from the sample is lower. However, even the background coming from the labeled structure itself, or from diffusing PAINT probes, can be problematic. This is especially true when imaging thicker samples and when imaging in 3D using long-range PSFs (as will be discussed in more detail in the “Single-Molecule Tracking and Super-Resolution Imaging in 3d” section). Therefore, reducing this background is critical to enable and improve imaging in these situations. For DNA-PAINT imaging, important recent advances, such as the development of fluorogenic probes (Chung et al., 2020), have been made to reduce the background coming from the diffusing unbound fluorophores. An additional and complementary approach to mitigate the issue with high fluorescence background is to use LSFM to optically section the sample and in that way improve the localization precision.

Improving the signal can be done by selecting fluorophores that are bright and yield many photons. For SPT, the fluorophore should also be small enough to not obstruct the motion of the tracked molecule and photostable to allow for long track lengths. In addition, it should be live-cell compatible and either genetically encoded or cell-membrane permeable unless tracking is done in membranes. The labeling should also be specific to the target. Fluorescent proteins fulfill many of these criteria and have revolutionized live-cell imaging (Chalfie, 2009; Shimomura, 2009; Tsien, 2009; Kremers et al., 2011). However, they are typically not as bright or photostable as synthetic dyes, which reduces the localization precision. Fluorescent proteins also require transfection and the expression of fluorescent proteins may perturb cell function. Synthetic dyes are generally brighter and more photostable than fluorescent proteins, but they can yield higher background due to unspecific binding. They also often require fixation and permeabilization for labeling of structures inside cells, and the labeling efficiency can be limited. Quantum dots provide bright labels, but their size and complex blinking behavior can limit their applicability. For SR imaging, the fluorophores should also be bright to improve the localization precision, but they must allow for control of the on/off state. In PAINT approaches, the on/off fraction is controlled *via* binding kinetics and fluorophore concentration rather than by the photophysics of the fluorophore, which is why the requirements on the fluorophores for PAINT imaging are less stringent than for the other techniques. Here, bright fluorophores can be imaged over longer exposure times to increase the number of photons collected and yield excellent localization precision, at the cost of increased imaging time. Many SR applications do not require live samples, so here fluorophores that are not cell membrane permeable could be used if the cell is permeabilized before labeling. Large efforts are continuously being made to improve parameters of fluorophores for SPT and SR imaging such as brightness, photostability, excitation and emission wavelengths, blinking and activation properties, cell permeability, and labeling specificity (Dempsey et al., 2011; Chozinski et al., 2014; Grimm et al., 2015, 2016a,b), so researchers should carefully select optimal fluorophore and imaging conditions for their specific applications.

## Single-Molecule Tracking and Super-Resolution Imaging in 3D

Given that 3D information is crucial for a complete understanding of biological specimens, modes of imaging that enable the acquisition of both lateral and axial information are invaluable. Confocal microscopy can provide excellent contrast and can generate 3D images through stacking of multiple *z*-planes, but it is inherently diffraction limited in all dimensions. Because of its confocal scanning nature, it also suffers from a limited temporal resolution on the order of seconds, and high peak powers increase the risk of photobleaching and photodamaging the sample. Fast 3D SPT has been achieved using confocal active-feedback approaches, such as orbital imaging (Levi et al., 2005; Katayama et al., 2009) and TSUNAMI (Perillo et al., 2015), where multiple confocal laser beams trace orbits around the tracked particle. An alternative active-feedback approach is based on a fast-scanning single confocal spot combined with a nanopositioner to keep the molecule within the 3D scanning region (Hou et al., 2019). The extension of this method to single molecules has historically been limited by piezoelectric response time and the number of photons detected from the single molecules. However, a recently developed method termed 3D Single Molecule Active Real-time Tracking (3D-SMART) has optimized these parameters to yield 3D tracking of single molecules with excellent temporal resolution over extended times (Hou et al., 2020). MINFLUX (Balzarotti et al., 2017), is another scanning approach that has recently been extended for 3D tracking and SR imaging. In MINFLUX, a fluorophore targeted by a doughnut-shaped beam will fluoresce more intensely the further it is from the center of the doughnut beam, which can be used to precisely determine the position of the fluorophore. MINFLUX has been used for SR imaging of the post-synaptic protein PSD-95 with essentially isotropic 3D resolution of 2–3 nm (Gwosch et al., 2020). This method facilitates work with very low photon counts and can achieve better spatial precision than other single-molecule localization techniques, which opens up possibilities for very detailed studies of synaptic structures and dynamics in the future. However, all these scanning methods have limitations when it comes to the number of particles that can be tracked or imaged in parallel.

Biplane (Toprak et al., 2007; Juette et al., 2008; Ram et al., 2008) and multiplane (Ram et al., 2012; Abrahamsson et al., 2013; Chen J. et al., 2014; Knight et al., 2015; Smith et al., 2015) imaging are wide-field approaches based on splitting the detected light into two or more light paths with different optical path lengths before they are imaged on a camera. This allows for the detection of the 3D position of many individual molecules in parallel. However, care has to be taken to balance the axial range used and the required spatial or temporal resolution, as the signal is weakened by splitting the light into multiple planes. Two-color biplane imaging combined with spectral demixing has been used to image nanostructures in 3D in hippocampal neurons, including β-tubulin, β2-spectrin, β4-spectrin, and AnkG in axons and Homer1 and Bassoon at the synapse (Winterflood et al., 2015). Biplane imaging has also been paired with advanced statistical analysis to determine the stoichiometry of and distance between the synaptic vesicle proteins synapsin and vesicular glutamate transporters (Lagache et al., 2018).

**Figure 4:**
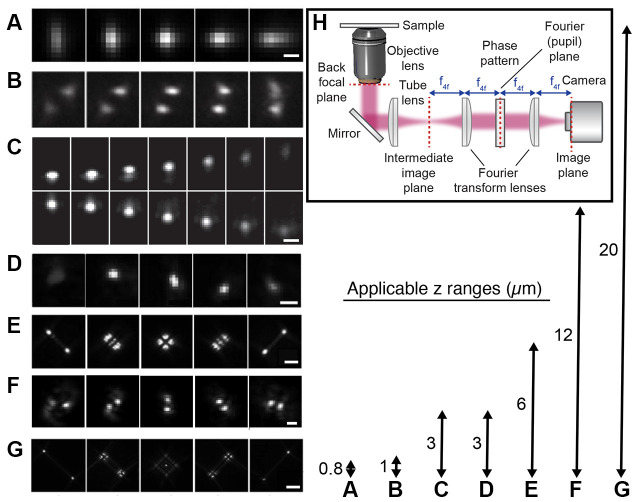
Engineered point spread functions (PSFs) for single-molecule localization allowing tracking and super-resolution imaging in 3D. The arrows (right) represent both the available axial ranges and the range over which the different PSFs were imaged, except in the case of the DH PSF that was imaged over a 3 μm axial range. **(A)** Astigmatic (Huang et al., 2008b). The scale bar is 0.5 μm. Reprinted from Huang et al. (2008b). Reprinted with permission from AAAS. **(B)** Phase ramp (Baddeley et al., 2011). Figure reprinted by permission from Springer Nature: Springer Nature, Nano Research (Baddeley et al., 2011), copyright 2011. **(C)** Accelerating beam (Jia et al., 2014). The scale bar is 1 μm. Figure reprinted by permission from Springer Nature: Springer Nature, Nature Photonics (Jia et al., 2014), copyright 2014. **(D)** Corkscrew (Lew et al., 2011). Reprinted with permission from Lew et al. (2011) © The Optical Society. **(E)** Tetrapod (Shechtman et al., 2015). The scale bar is 2 μm. Reprinted from Shechtman et al. (2015) with permission from the American Chemical Society (https://pubs.acs.org/doi/10.1021/acs.nanolett.5b01396). Further permissions related to the material excerpted should be directed to the ACS. **(F)** Double-helix (DH; Pavani et al., 2009). The scale bar is 2 μm. Reprinted with permission from Pavani et al. (2009). **(G)** Tetrapod (Shechtman et al., 2015). The scale bar is 5 μm. Reprinted from Shechtman et al. (2015) with permission from the American Chemical Society (https://pubs.acs.org/doi/10.1021/acs.nanolett.5b01396). Further permissions related to the material excerpted should be directed to the ACS. **(H)** The optical design used for PSF engineering was implemented with a transmissive dielectric phase mask to modulate the shape of the PSF.

**Figure 5:**
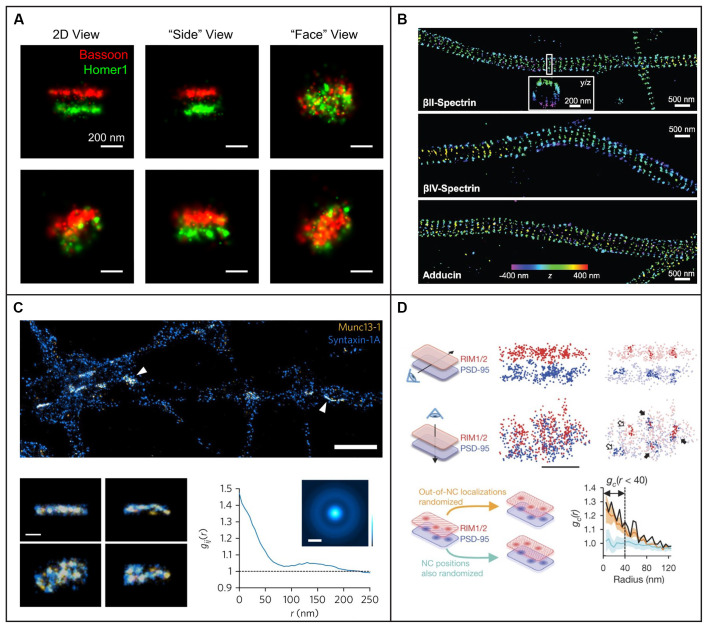
Examples of 3D single-molecule super-resolution imaging in neurons and synapses. **(A)** STORM with astigmatism was used to image pre- and postsynaptic scaffolding proteins Bassoon and Homer1 in 3D, providing information about the shape of the presynaptic active zone and postsynaptic density from different angles for two different pre- and postsynaptic pairs in the top and bottom row, respectively. The scale bars are 200 nm. Figure reprinted with permission from Dani et al. (2010). **(B)** STORM with astigmatism was used to image and analyze the 3D organization of cytoskeletal protein spectrin and actin-capping protein adducin, revealing their periodic axon-surrounding structure. The scale bars are 500 nm. Figure reprinted from Xu et al. (2013). Reprinted with permission from AAAS. **(C)** STORM with astigmatism was used to image multiple views of Munc13-1 (yellow) assemblies and syntaxin-1A (blue) at the active zone. The graph illustrates pair correlation analysis of the STORM datasets, demonstrating nanoscale co-clustering of Munc13-1 and syntaxin-1A. The scale bar is 2 μm in the top panel and 200 nm in the bottom panel. Reprinted by permission from Springer Nature: Springer Nature, Nature Neuroscience, (Sakamoto et al., 2018), copyright 2018. Reprinted with kind permission from Springer. **(D)** Light sheet illumination combined with two-color 3D-STORM was used for imaging of synaptic RIM1/2 and PSD-95. The trans-synaptic alignment of protein nanoclusters in the active zone and the PSD are shown both with top and side views. Closed arrows indicate the trans-synaptic alignment and open arrows correspond to non-aligned nanoclusters. In the lower panels a paired cross-correlation function is shown for RIM1/2 and PSD-95 distributions in two different simulated conditions: first, for randomly distributed nanoclusters (light blue trend), and second, for a random selection of molecules outside the originally measured nanocluster positions (orange trend). The scale bar is 200 nm. Reprinted by permission from Springer Nature: Springer Nature, Nature, (Tang et al., 2016), copyright 2016.

An alternative wide-field approach is to use engineered PSFs (for a review, see e.g., von Diezmann et al., 2017), where the axial (*z*) position of the emitter is encoded directly in the shape of the PSF on the camera (Figure 4). This is accomplished by modifying the phase pattern of the emitted light in the Fourier plane of the microscope and allows for scan-free wide-field 3D detection of emitters with excellent precision by the addition of a small number of optical elements to a standard fluorescence microscope. A common approach is to introduce a weak cylindrical lens in the emission light path to create an astigmatic PSF (Kao and Verkman, 1994; Huang et al., 2008a; Spille et al., 2012; Li et al., 2013; Izeddin et al., 2014). An early use of astigmatic PSFs for 3D SR imaging of the synapse involved STORM imaging of the architecture and distance between 10 protein components of the presynaptic active zone and the postsynaptic density in brain tissues (Dani et al., 2010; Figure 5A). Astigmatism in combination with STORM was also used to discover that axons are wrapped in evenly-spaced periodic structures called the membrane-associated periodic skeleton, composed of actin, spectrin, and other related proteins (Xu et al., 2013; Figure 5B). These studies have also been extended to map the membrane-associated periodic skeleton in axons, dendrites, and soma of neurons at different developmental stages (Zhong et al., 2014; Han et al., 2017). Astigmatism and dSTORM have also been used to determine the location of the transmembrane protein assembly γ-secretase, an enzyme linked to Alzheimer’s disease (Schedin-Weiss et al., 2016), as well as to identify a mechanism for controlling synaptic weight through imaging of Munc13-1 supramolecular assemblies (Sakamoto et al., 2018; Figure 5C). More recently, astigmatism was employed to map protein distributions and arrangements within a calyx of Held synapse through multiplexed dSTORM imaging (Klevanski et al., 2020). Astigmatism has also been combined with PALM and STORM for two-color 3D SR imaging to characterize the ultrastructure of inhibitory synapses and to count scaffold proteins and receptor binding sites (Specht et al., 2013). In addition, astigmatism has been used with two-color STORM for 3D SR imaging together with EM and STED to determine the spatial distribution of proteins EphB2 and SynCAM in relation to the postsynaptic density, which revealed that SynCAM 1 shapes the cleft edge, while EphB2 is enriched deeper into the postsynapse (Perez de Arce et al., 2015), as well as for imaging of the distribution of presynaptic proteins in relation to the postsynaptic scaffolding protein PSD-95, which revealed trans-synaptic alignment of the distributions (Tang et al., 2016; Figure 5D).

PSF engineering has also been used to create more complex PSFs with various axial ranges between 0.8 and 20 μm, including the bisected pupil (Backer et al., 2014), self-bending (Jia et al., 2014), corkscrew (Lew et al., 2011), double helix (DH; Pavani et al., 2009; Thompson et al., 2010; Backlund et al., 2014; Gustavsson et al., 2018a; Bennett et al., 2020), and tetrapod PSFs (Shechtman et al., 2014, 2015, 2017; Weiss et al., 2020). The desired phase pattern can be implemented using transmissive dielectric phase masks, a liquid crystal spatial light modulator (SLM), or a deformable mirror. Transmissive dielectric masks allow for the implementation of any type of phase pattern with excellent photon efficiency. However, one mask is required for each type of PSF, axial range, and wavelength range used. An SLM also allows for the implementation of any type of phase pattern; the choice of pattern is flexible and can be easily and rapidly updated. However, an SLM can only modulate one polarization direction of the emitted light, which means that either half of the light has to be discarded, at the cost of reduced localization precision, or more elaborate optical designs have to be implemented to recover the other polarization direction (Backlund et al., 2012). The deformable mirror consists of a continuous membrane and is therefore only suited for smoothly varying phase patterns. For these types of phase patterns, the deformable mirror can be easily and rapidly updated to facilitate various axial ranges and wavelengths and offers excellent photon efficiency.

Longer-range engineered PSFs have not yet been extensively implemented for imaging in cultured neurons or in brain tissues, but they hold great promise for addressing questions about 3D molecular dynamics and nanoscale morphology in these types of samples. Just like in the case of localization microscopy in 2D, the spatiotemporal resolution that can be achieved for 3D localization using engineered PSFs depends on the signal-to-background ratio between the signal from the fluorophore and the background fluorescence from the rest of the cell. The footprint of engineered PSFs on the camera is larger, which means that the signal photons are spread over more pixels. Since imaging in thick samples, such as entire cells or tissues, typically results in high fluorescence background, methods to improve the signal-to-background ratio are critical to enable and improve 3D single-molecule imaging in these situations. Combining engineered PSFs with LSFM for optical sectioning of thick samples is thus a promising route to solve these problems which can pave the way for new discoveries in neuroscience.

## Light Sheet Illumination Strategies for Improved Wide-Field Detection of Single Molecules in 3D

LSFM has revolutionized large-scale imaging of brain tissue and neural networks, but its optical sectioning capability also greatly benefits SPT and single-molecule SR imaging (for more extensive reviews on the marriage of single-molecule approaches with LSFM, see e.g., Power and Huisken, 2017; Gustavsson et al., 2018b). When merging single-molecule approaches with LSFM, a high numerical aperture (NA) detection objective is ideal to capture as many photons as possible emitted from the individual fluorophores to improve the detected signal, as well as a high-NA illumination objective to create a thin light sheet to reduce out-of-focus background fluorescence, photobleaching, and photodamage. However, high-NA objectives have short working distances and large physical profiles, which prevent concomitant use in most LSFM designs. For whole-cell or tissue imaging, it is desirable that fluorophores can be excited all the way down to the coverslip, but this is difficult to achieve using SPIM-like approaches with high-NA objectives. Several different approaches have therefore been designed to address these challenges.

An early implementation for single-molecule imaging utilized the conventional SPIM approach with a cylindrical lens and a long working distance illumination objective to achieve high contrast for SPT in *C. tentans* larvae (Ritter et al., 2010). However, due to the use of a cylindrical lens which forms a light sheet with a Gaussian profile that diverges away from the focus, this light sheet could not be used to excite fluorophores all the way down to the coverslip because of aberrations in the light sheet at the interface between the sample chamber wall and the coverslip would have been introduced. This method was thus limited to imaging 100–200 μm above the coverslip, which prevents imaging of individual adherent mammalian cells or tissues.

**Figure 6:**
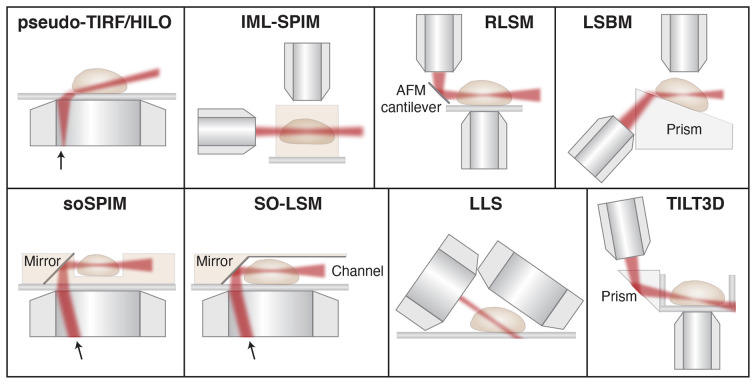
Schematic showing different light sheet approaches developed for improved single-molecule tracking and super-resolution imaging. Pseudo- TIRF/HILO, highly inclined and laminated optical sheet (Tokunaga et al., 2008); IML-SPIM, individual molecule localization with selective-plane illumination microscopy (Cella Zanacchi et al., 2011); RLSM, reflected light sheet microscopy (Gebhardt et al., 2013); LSBM, light-sheet Bayesian microscopy (Hu et al., 2013); soSPIM, single-objective SPIM (Galland et al., 2015); SO-LSM, single-objective light-sheet microscopy (Meddens et al., 2016); LLS, lattice light-sheet (Chen et al., 2014); TILT3D, tilted light sheet microscopy with 3D PSFs (Gustavsson et al., 2018a). Illustrations are not to scale. Figure adapted with permission from Gustavsson et al. (2018b) © The Optical Society.

One convenient way to circumvent the issue of steric hindrance between two high-NA objectives is to use a single high-NA objective for both fluorescence detection and the formation of an oblique light sheet for excitation. Multiple different implementations of this type of pseudo-TIRF illumination have been developed, including highly inclined and laminated optical sheet (HILO; Tokunaga et al., 2008), variable-angle epifluorescence microscopy (VAEM; Konopka and Bednarek, 2008), and methods that address the resulting angle mismatch between the light sheet plane and the image plane by rotating the image plane (Dunsby, 2008; Bouchard et al., 2015; Maioli et al., 2016) or by adding a second detection objective perpendicular to the light sheet plane (Theer et al., 2016; Figure 6). However, the latter methods come at the cost of increased complexity and a lower effective NA in the detection path, which reduces the detected signal. Overall, single-objective oblique light sheet methods offer a convenient approach for optical sectioning using a conventional epi-fluorescence microscope. However, these light sheets are generally relatively thick, the thickness and intensity of the light sheet depend on the incident angle, and the FOV is limited due to the non-planarity between the light sheet and image planes unless one of the approaches above is implemented to address this issue.

Another approach termed light-sheet Bayesian microscopy (LSBM) resembles the single-objective oblique methods, but here a separate illumination objective is used together with a Pellin-Boca prism to direct the light sheet along the image plane (Hu et al., 2013, 2016). In this design, the entire sample is angled instead of the illumination and image planes, which circumvents the issue with non-planarity between the light sheet and image planes. Since the resulting light sheet is still relatively thick, the authors paired this illumination scheme with Bayesian bleach-and-blink (3B) image reconstruction of PALM/STORM data (Cox et al., 2012; Rosten et al., 2013) to better localize molecules in dense regions. This design also utilizes a somewhat lower-NA detection objective of NA 1.0, which reduces the achievable localization precision. Furthermore, the addition of the prism pathway and the custom-made sample holder adds complexity and prevents direct addition to a conventional microscope.

The first approach that utilized SPIM for single-molecule SR imaging is termed individual molecule localization with selective plane illumination (IML-SPIM; Cella Zanacchi et al., 2011). Here the thickness of the light sheet can be tuned depending on the illumination objective and the light sheet parameters are not coupled with the light sheet position. Using this approach together with PALM and astigmatism, the authors demonstrated 3D single-molecule SR imaging in up to 150 μm thick samples. Because the method relies on conventional SPIM, the sample must be mounted in an agarose gel away from the coverslip, which is not compatible with studies of adherent, mammalian cells or samples positioned on conventional coverslips. However, this method demonstrates that 3D single-molecule imaging can be achieved even far away from the coverslip with a relatively simple setup and a NA 1.1 detection objective.

Multiple different methods have then been developed to circumvent the restrictions posed by SPIM and oblique light sheet approaches for SPT and SR imaging. In reflected light sheet microscopy (RLSM), a second illumination objective is mounted vertically and used to form a light sheet that is then redirected parallel with the image plane by reflection off of a polished atomic force microscopy (AFM) cantilever positioned directly adjacent to the sample (Gebhardt et al., 2013). A similar approach has also been developed that uses micro-prisms on the coverslip rather than an AFM cantilever for reflection (Greiss et al., 2016). These methods utilize high-NA objectives for both illumination and detection, which allows for the formation of very thin light sheets and excellent detection efficiency. However, they suffer from a gap of about 2 μm just above the coverslip that is inaccessible to the light sheet due to the Gaussian beam profile.

Similar approaches based on reflection to redirect the light sheet to overlap with the image plane have also been developed using only a single objective. In single-objective SPIM (soSPIM), the light sheet exits the objective lens vertically and offset from the center of the FOV, and is then reflected using a micro-mirror mounted at 45° in a custom-made sample chamber, similar to an inverted RLSM design (Galland et al., 2015). In single-objective light-sheet microscopy (SO-LSM), the light sheet is instead directed along the image plane using a microfluidic chamber with reflective side walls angled at 45° (Meddens et al., 2016). Both of these methods allow for the formation of very thin light sheets and detection with high efficiency using a high-NA objective on a conventional epi-illumination microscope. In these designs, the illumination and detection optics are coupled, so axial scanning requires synchronous translation of the objective lens or sample stage, translation of the light sheet beam at the mirror, and defocusing of the light sheet beam to keep the beam waist in the center of the FOV. This has been achieved using a beam-steering unit and an electrically tunable lens to translate the light sheet axially and along the optical axis in the image plane. In soSPIM, the sample is placed in raised wells and in SO-LSM the sample is mounted in agarose gel to suspend the sample above the coverslip to allow for imaging throughout the sample. Although these optical and electronic designs are relatively complex compared to some other light sheet designs and the custom-made sample chambers require sophisticated fabrication approaches, they have successfully been implemented for 3D SPT and SR imaging using astigmatism.

Conventional Gaussian light sheets are inherently limited by the diffraction-based trade-off between the thickness of the light sheet at its beam waist and the depth-of-focus over which it remains thin. Bessel and Airy beams are examples of invariant beams that have the useful property of staying focused over a long distance along the propagation direction (Durnin et al., 1987; Fahrbach and Rohrbach, 2010; Vettenburg et al., 2014; Yang et al., 2014), meaning a narrow 1D beam can be scanned to form a thin plane of excitation light. These beams are also self-healing (Bouchal et al., 1998; Fahrbach and Rohrbach, 2010; Fahrbach et al., 2010; Nylk et al., 2016; Zhang et al., 2016), which is valuable when propagating through scattering media such as neuronal tissue (Fahrbach and Rohrbach, 2012; Nylk et al., 2016). One issue with Bessel and Airy beams is that they have prominent side lobes which excite fluorophores outside of the light sheet plane. A solution to this problem was developed with the invention of lattice light sheet (LLS) microscopy (Chen B.-C. et al., 2014), where Bessel beams in a linear array are spaced with a specific periodicity such that the beams interfere constructively in the main lobes and destructively elsewhere to greatly reduce the contributions from the side lobes. This design can be used either in an SR-SIM mode due to its structured illumination, or it can be dithered to create a very thin and uniform light sheet. LLS has been demonstrated for 3D SPT and single-molecule SR microscopy through pairing with astigmatism for e.g., PALM imaging of the entire nuclear lamina (Chen B.-C. et al., 2014), PAINT imaging of ER structures (Nixon-Abell et al., 2016), and multicolor PALM and PAINT imaging of DNA, the nuclear lamina, and intracellular membranes (Legant et al., 2016). LLS provides a powerful tool for SPT and single-molecule SR imaging and it can be used for imaging of entire adherent mammalian cells as well as larger samples. However, steric hindrance between the objectives and between the detection objective and the coverslip prevent the use of commercially available higher-NA detection objectives. In the original study, the illumination objective was custom-designed and the NA of the detection objective was limited to 1.1. The optical and electronic complexity and cost might also prevent implementation in many laboratories.

More recently, our group developed a method that combines a tilted light sheet with engineered PSFs (TILT3D; Gustavsson et al., 2018a). Here the light sheet is formed by a cylindrical lens, focused by a long working distance illumination objective, and reflected into the sample at an angle by a prism or mirror. This approach requires only simple optics to form the light sheet and the tilt allows for imaging of entire adherent samples all the way down to the coverslip. By pairing this tilted light sheet illumination with long-range PSFs, there is no need to create a very thin light sheet using complicated optics and electronics, since the 3D position of individual molecules will be detected throughout the entire volume excited by the light sheet and determined by the shape of the PSFs on the camera. TILT3D allows for flexible yet simple 3D SPT and single-molecule SR imaging in 3D over a user-defined axial range using conventional coverslips, a conventional epi-fluorescence microscope, and a high-NA detection objective. In the original implementation, a 2.1 μm thick light sheet was used together with an NA 1.4 detection objective and a two-channel detection module where a DH PSF was used for single-molecule imaging with high precision and a tetrapod PSF was used for detection of fiducial beads for drift correction over a large axial range (Gustavsson et al., 2018a). This design has allowed for 3D dSTORM imaging of e.g., mitochondria (Gustavsson et al., 2018a), the entire nuclear lamina (Gustavsson et al., 2018a), and sugars in the glycocalyx in cancer cells (Möckl et al., 2019a). The optical sectioning would be improved further by using thinner light sheets, and the design is compatible with the implementation of Bessel beam light sheets, but at the cost of increased complexity. Nevertheless, even the original design improved the signal-to-background ratio up to five-fold throughout the mammalian cells compared to epi-illumination. By matching the axial range of the used PSF to the thickness and tilt of the light sheet over the used FOV, TILT3D provides a very simple and effective platform for 3D SPT and single-molecule SR imaging with high precision, and it has great potential to be a useful tool for improved imaging in neurons and neuronal tissues in the future.

## Applications of Light Sheet Single-Molecule Imaging in Neurons and Synapses

Combining SPT and SR techniques with LSFM allows for improved investigation of the dynamics and organization of single molecules in neurons. Oblique LSFM was combined with sptPALM to improve the localization precision and live-cell compatibility when studying actin within dendritic spines (Frost et al., 2010). It was also used with sptPALM for tracking and classification of the dynamics of CaMKII in different regions of dendritic spines (Lu et al., 2014), and for tracking of cortactin in the investigation of the role of Shank proteins as links between the synapse and the actin cytoskeleton (MacGillavry et al., 2016). Oblique LSFM illumination has also been combined with live-cell PALM for imaging of the subsynaptic distribution of PSD-95 in rat hippocampal neurons (MacGillavry et al., 2013). In addition, oblique LSFM has been implemented for two-color dSTORM and PALM with astigmatism for pairwise 3D SR imaging of the synaptic proteins RIM1/2, PSD-95, GKAP, and Shank to reveal trans-synaptic nanocolumns that aligned neurotransmitter release to receptors (Tang et al., 2016; Figure 5D). Bessel light sheets combined with the spontaneously blinking fluorophores HMSiR (Uno et al., 2014) have been used to super-resolve the structure of dopaminergic neurons in the adult *Drosophila* brain (Chu et al., 2019). More recently, a Bessel light sheet together with astigmatism was used to obtain 3D super-resolved reconstructions of microtubule networks in the primary neurons of rat pups for large FOVs (Lu et al., 2019).

Moreover, LSFM has been combined with Super-resolution Optical Fluctuation Imaging (SOFI; Dertinger et al., 2009) and Super-Resolution Radial Fluctuations (SRRF; Gustafsson et al., 2016), which utilize computational analysis of temporal (SOFI and SRRF) and radial (SSRF) intensity fluctuations of fluorophores in acquired image series to generate a super-resolved reconstruction. Although these methods do not technically localize individual molecules and generally do not achieve the same resolution as single-molecule localization techniques, they can be useful because of their simplicity, ability to image densely labeled samples rapidly, and their low-excitation intensity requirements. Recently, LSFM has been successfully combined with SRRF and 3B analysis to image neurons in the *Drosophila* brain (Chen et al., 2020). High-contrast imaging was achieved using a 700 nm thin Bessel light sheet, and reconstructions from these slices were combined to form a 3D rendering. In a very recent study, the combination of LSFM with SOFI (LS-SOFI) achieved rapid SR analysis of neuronal structures and synaptic proteins (Mizrachi et al., 2020).

3D single-molecule LSFM is a comprehensive method that maintains the benefits of conventional fluorescence microscopy while improving precision, resolution, and live-cell compatibility. This allows for high-contrast gentle SPT in living cells and nanoscale visualization of synaptic structures in neurons which offers numerous opportunities for future discoveries in cell biology and biomedical research. Extending single-molecule LSFM for imaging over large areas may also provide insights into how single-molecule interactions affect the brain as a whole.

## Discussion

The inherent optical sectioning capability of LSFM that offers reduced fluorescence background, photobleaching, and photodamage is particularly well-suited for single-molecule imaging in thick or sensitive samples. Since the developments of single-molecule SR imaging and LSFM over a decade ago, there have been continuous developments in both these techniques to improve the speed, sensitivity, accuracy, imaging depth, and live-cell compatibility.

One LSFM approach that allows for both excellent sectioning and imaging deeper into the sample is to use light sheets based on propagation invariant beams, such as Bessel and Airy beams. Their non-diffractive (Durnin et al., 1987) and self-healing (Bouchal et al., 1998) properties, as well as their larger depth of field when compared to regular Gaussian light sheets, which diverge quickly after focusing, make them particularly useful for imaging in thick, scattering samples, such as neuronal tissue (for a review, see e.g., Corsetti et al., 2019). The choice of light sheet approach should be made considering both the needed optical sectioning ability and the experimental implementation requirements, where invariant beams allow for thinner light sheets, but at the price of increased complexity and cost compared to Gaussian light sheets.

Neurons and neuronal tissues cause aberrations and scattering of the light that affects both the illumination and detection pathways. These aberrations will reduce the optical sectioning and penetration depth for LSFM and reduce the collection efficiency and the quality of the PSF for single-molecule localization. Adaptive optics (AO) is an approach that can be used to counteract the effects of optics- and sample-induced aberrations to improve the characteristics of both the light sheet in the illumination pathway and the PSF in the detection pathway (Burke et al., 2015; Wang et al., 2015; Wilding et al., 2016; Ji, 2017). AO can be implemented using deformable mirrors or SLMs. AO thus has the possibility to improve the precision, accuracy, and useful imaging depth for single-molecule LSFM imaging.

SPT and SR LSFM in neurons and synapses will also benefit from the ongoing developments of fluorophores with improved photostability, brightness, excitation and emission wavelengths, blinking and activation properties, labeling specificity, cell membrane permeability, and live-cell compatibility. Such developments include far-red fluorophores that can be actively controlled in live-cell compatible conditions (Lukinavičius et al., 2013, 2014; Grimm et al., 2016a,b, 2017; Bucevičius et al., 2020), as well as fluorogenic DNA-PAINT probes (Chung et al., 2020) and improvements to DNA-PAINT docking strands and imaging buffer (Civitci et al., 2020) for faster imaging with improved signal-to-background ratio.

As previously mentioned, MINFLUX is an emerging method that allows for imaging with nanometer precision (Balzarotti et al., 2017; Gwosch et al., 2020). Although the widespread application has so far been limited due to the technical complexity of the method, it has the potential to address a range of questions related to synaptic nanoscale structure and dynamics in the future.

Expansion microscopy (ExM) is a method of physically increasing the size of cells and tissues using an isotropically expandable gel (Chen F. et al., 2015; Wassie et al., 2019). ExM has proven useful for nanoscale imaging of neuronal samples, including imaging of the synaptic proteins Homer and Bassoon (Chozinski et al., 2016), actin filament organizations in dendrites and postsynaptic spines (Park et al., 2020), the morphology of gonadotropin-releasing hormone neurons (Wang et al., 2020), and the neuronal cytoskeleton (Jurriens et al., 2021). ExM has been paired with LLS to achieve a nanoscale resolution to uncover the organization of proteins and neural circuits in mouse brain samples and in the entire *Drosophila* brain (Bürgers et al., 2019; Gao et al., 2019). ExM has also been combined with single-molecule SR approaches like dSTORM (Xu et al., 2019) and 3D dSTORM using astigmatism (Zwettler et al., 2020), but so far such applications for neuronal or synaptic imaging are limited. In ExM-dSTORM, care must be taken as the fluorophores can be degraded if labeling is done before the sample preparation, and conventional blinking buffers can shrink the gel if it is not stabilized. Another option is to use fluorophores that will spontaneously blink in a gel-compatible buffer, such as silicon rhodamines (Zwettler et al., 2020). ExM has the potential to be combined with both LSFM and 3D single-molecule SR approaches for future studies of neuronal samples and synapses. However, the researcher must be careful to ensure that the chemical linking to the gel does not cause distortions of the structure of interest. ExM is also limited to imaging of fixed samples and cannot be used for live-cell studies.

Recently, important advances have also been made to improve SPT and single-molecule SR imaging in densely labeled samples using deep learning (for a review, see e.g., Möckl et al., 2020a). These developments include using neural nets to optimize and localize engineered PSFs faster and for overlapping emitters (Nehme et al., 2018, 2020; Zhang et al., 2018), phase retrieval of aberrations, and background correction (Paine and Fienup, 2018; Möckl et al., 2019b, 2020b; Saha et al., 2020). The next steps in this field are to standardize controls to ensure the validity of the analysis, as well as to make these approaches more easily accessible and comparable for a wide range of users. The combination of LSFM with 3D single-molecule localization microscopy provides a powerful tool for tracking single-molecule dynamics and imaging of nanoscale structures in neuronal synapses. Continued developments in these fields will further improve not only the achievable precision and resolution but also the live-cell compatibility and the ease of implementation of the methods. In the future, this will hopefully lead to improved understanding and new discoveries about the molecular mechanisms at work all the way from within individual synapses to the entire brain.

## Author Contributions

GG, TN, NS, SV-H, and A-KG wrote the manuscript. All authors contributed to the article and approved the submitted version.

## Conflict of Interest

The authors declare that the research was conducted in the absence of any commercial or financial relationships that could be construed as a potential conflict of interest.

## Publisher’s Note

All claims expressed in this article are solely those of the authors and do not necessarily represent those of their affiliated organizations, or those of the publisher, the editors and the reviewers. Any product that may be evaluated in this article, or claim that may be made by its manufacturer, is not guaranteed or endorsed by the publisher.
